# In-vivo optical imaging in head and neck oncology: basic principles, clinical applications and future directions

**DOI:** 10.1038/s41368-018-0011-4

**Published:** 2018-03-18

**Authors:** Chenzhou Wu, John Gleysteen, Nutte Tarn Teraphongphom, Yi Li, Eben Rosenthal

**Affiliations:** 1 State Key Laboratory of Oral Diseases & National Clinical Research Center for Oral Diseases & Department of Head and Neck Oncology, West China Hospital of Stomatology, Sichuan University, Chengdu, China; 2 Department of Otolaryngology, University of Tennessee Health Science Center, 38163 Memphis, TN, USA; 3 Department of Otolaryngology and Radiology, Stanford University, 94305 Stanford, CA, USA

## Abstract

Head and neck cancers become a severe threat to human’s health nowadays and represent the sixth most common cancer worldwide. Surgery remains the first-line choice for head and neck cancer patients. Limited resectable tissue mass and complicated anatomy structures in the head and neck region put the surgeons in a dilemma between the extensive resection and a better quality of life for the patients. Early diagnosis and treatment of the pre-malignancies, as well as real-time in vivo detection of surgical margins during en bloc resection, could be leveraged to minimize the resection of normal tissues. With the understanding of the head and neck oncology, recent advances in optical hardware and reagents have provided unique opportunities for real-time pre-malignancies and cancer imaging in the clinic or operating room. Optical imaging in the head and neck has been reported using autofluorescence imaging, targeted fluorescence imaging, high-resolution microendoscopy, narrow band imaging and the Raman spectroscopy. In this study, we reviewed the basic theories and clinical applications of optical imaging for the diagnosis and treatment in the field of head and neck oncology with the goal of identifying limitations and facilitating future advancements in the field.

## Medical imaging: Finding the edge

New imaging technologies hold promise for early detection and improved treatment of head and neck cancers, the sixth most common type of cancer worldwide. The first line of treatment is surgery, but complete tumor removal can be difficult when operating on this sensitive region. Improving methods for early detection and clear visualization of tumor edges could help minimize loss of healthy tissue. Eben Rosenthal at Stanford University in California and Yi Li at the West China School of Stomatology in Chengdu City reviewed new technologies, many of which use fluorescence to identify tumor tissue. Some techniques even allow real-time visualization of tumor edges in the operating theatre. The researchers discuss the applications and limitations of these new technologies and highlight promising future directions, including increasing the commercial availability and decreasing the size of the instruments.

## Introduction

Head and neck cancers represent the sixth most common malignancy worldwide with ~529 500 new patients diagnosed annually and are responsible for 3.6% of cancer-specific deaths.^1^ More than 90% of head and neck cancers are squamous cell carcinomas (HNSCC) that arise from the mucosal surfaces of the oral cavity, oropharynx, and larynx. HNSCC accounts for 5%–10% of all new cancer cases in the North America and Europe, although worldwide there are geographic variations in the incidence and anatomic distribution. In high-risk countries (i.e., India, Sri Lanka, Bangladesh, and Pakistan), oral cavity squamous cell carcinoma (OSCC) is the most common cancer in men and the third most common cancer in women.^2^ More than 50% of patients with advanced OSCC survive <1 year from their time of diagnosis, owing to both locoregional and distant failure.^3^

Current imaging strategies commonly used for cancer detection and pretreatment planning are based on anatomic or metabolic changes in the tissue. Recent advances in optical hardware and reagents have provided unique opportunities for real-time cancer imaging in the clinic or operating room. Optical techniques have been widely used to detect early stage disease based on subtle surface changes associated with mucosal growth. These strategies have also been applied to the surgical setting where the parameters of the tumor have been better-defined using optical imaging.

Surgical resection with 1–2 cm margins is the primary treatment modality for OSCC and early stage oropharynx cancer; real-time in vivo detection of surgical margins during en bloc resection could be leveraged to minimize the resection of normal tissues. Margin analysis is traditionally performed by histological investigation of biopsy samples, although this method has some inherent disadvantages. First, it is a subjective method based on the experience and ability of the pathologist. Second, the malignant focus may be too small to be detected in the sectioning which can lead to sampling error and false negative diagnosis. And third, the frozen section of the surgical margin is time-consuming, often with substantial lapses in time between biopsy of the margin and the acquisition of the result. Re-excision of a positive margin after a delay can sometimes involve guesswork as to the exact location of the margin.

As the understanding of the biology and tumorigenesis of head and neck cancer has advanced, including identification of specific biomarker expression in tumor cells, upregulated metabolic activities, and the variations in tumor microenviroment, new diagnostic methods and instruments have developed. Optical imaging permits real-time diagnosis and margin discrimination, which would be most helpful to surgeons in the minimally invasive setting when physical cues like visualization and palpation are absent.^4^ Optical imaging or light based imaging techniques, uses specific properties of light to image anatomical or chemical characteristics of tissue. Analogous to many radiolabeled agents, imaging of optical contrast is performed using ligands conjugated to an optically active reporter to target a recognized disease biomarker.^5^ Optical imaging in the head and neck has been reported using autofluorescence imaging (AFI),^5^ targeted fluorescence imaging (TFI),^5^ high-resolution microendoscopy (HRME),^6^ narrow band imaging (NBI),^7^ and the Raman spectroscopy (RS).^8^ Besides these, other optical imaging modalities, such as optical coherence tomography, elastic scattering spectroscopy, confocal laser endomicroscopy, and confocal reflectance microscopy, have also been widely applied in the head and neck region and were nicely reviewed elsewhere.^9–11^ In this study, we specially reviewed the basic theories and clinical applications of AFI, TFI, HRME, NBI, and RS for the diagnosis and treatment of head and neck cancer with the goal of identifying limitations and facilitating future advancements in the field.

## Basic principle of fluorescence imaging

**Fig. 1:**
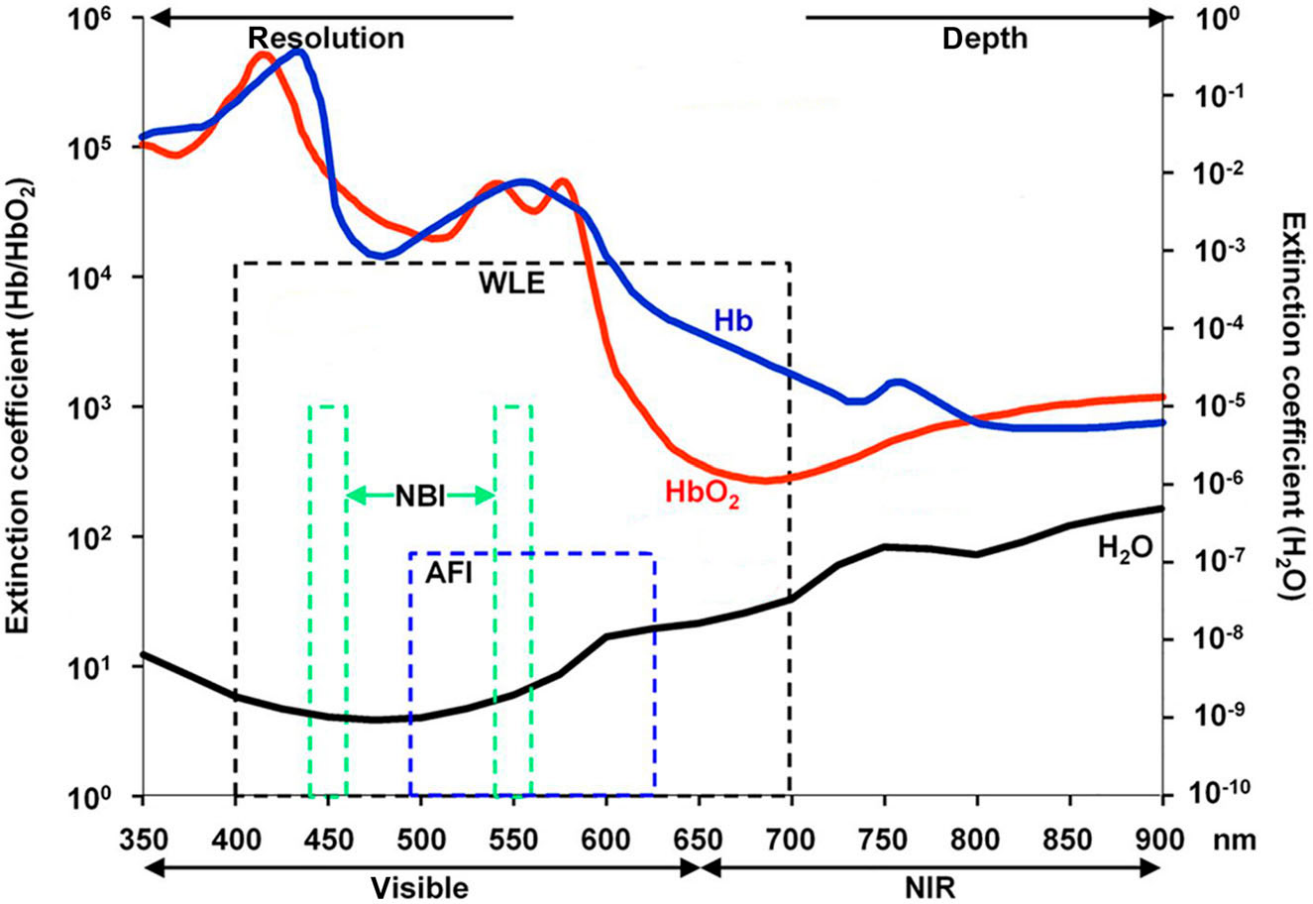
Optical properties of tissue and the different wavelength range of each fluorescent imaging devices. Image resolution improves with shorter wavelengths, and tissue penetration increases with longer wavelengths. Hemoglobin (Hb) dominates absorption of light in the visible (400–700 nm). Water absorption plays a small role in the infrared (>900 nm). The extinction coefficients for Hb (blue), oxyhaemoglobin (HbO_2_; red) and water (black) are shown. AFI, autofluorescence imaging; NBI, narrow band imaging; WLE, white-light endoscopy. Reprinted with permission from ref. ^16^ by BMJ Publishing Group Ltd. and Copyright Clearance Center

The basic principle of fluorescence imaging has been reviewed in detail previously.^12–14^ Briefly, the illumination light from a filtered light source (low-intensity excitation) or laser (high-intensity excitation) enters and travels through tissue to reach and be absorbed by the targeted fluorophores, which can be either endogenous (i.e., autofluorescence) or exogenous (i.e., injected fluorescein). Absorption of the photons causes an excited state of the fluorophore, which then re-emits photons as it returns to its ground state. The re-emission photons can be detected with a charged coupled device (CCD) camera that can provide color and fluorescence imaging, either separately or on an overlay pseudocolour image in real-time. The energy level transition between the excited state and ground state causes energy loss and results in a shift from shorter wavelengths (higher energy) of the absorption spectrum to longer wavelengths (lower energy) of the emission spectrum, known as the Stokes shift. This process lasts for a few nanoseconds depending on the fluorophore and is called the lifetime of fluorophore. Both the illumination and the emission light have to travel through tissues and are mainly affected by: (1) reflection of tissue surface; (2) refraction of tissue surface; (3) tissue scattering; and (4) absorption of light mainly by water, lipids (absorb light in the infrared range, >900 nm) and hemoglobin (absorb light in visible light spectrum, <600 nm). Since the absorption spectrums of water, lipids, and hemoglobin are non-overlapped, an optical imaging window exists in the near-infrared (NIR) spectrum (~650–900 nm) where the absorption coefficient of tissue is at a minimum. In addition, recent studies have shown that the extending into the longer wavelengths results in better tissue penetration because of less scattering, whereas image resolution improves with shorter wavelengths.^15,16^ Figure 1 displays the optical properties of tissues and the different wavelength range of each fluorescent imaging device.^16^

## Autofluorescence imaging (AFI)

### Basic theory

Autofluorescence is the natural fluorescence of endogenous fluorophores, primarily nicotinamide adenine dinucleotide (NADH) and flavin adenine dinucleotide (FAD), without the addition of any chemical substances.^17^ AFI is an imaging modality that helps visualize the autofluorescence spectrum of endogenous fluorophores. When normal tissues are illuminated by AFI devices with ultraviolet (200–400 nm) and visible light (400–600 nm) they emit fluorescent light, while neoplastic tissues actually appear darker compared with the healthy surroundings due to autofluorescence loss.^18^ The autofluorescence loss-of-neoplastic tissues is mainly caused by metabolism alterations and morphologic changes of the epithelial surface and underlying stroma.^19–21^ Specifically, increased numbers of nuclei and increased microvascularity leads to scattering and absorption of illumination light, and decreased content of the collagen matrix and elastin directly results in lower autofluorescence intensity.^17^ These neoplastic-related changes reduce the detectable autofluorescence signal, thus leading to autofluorescence loss.

Even though the AFI modalities are regarded as practical, cost-effective and non-invasive, they may suffer from an innate disadvantage: low specificity.^20,21^ The false positives are related to tissues with rich microvascularity causing scattering and autofluorescence loss, seen in granulation tissue, inflammation, and edema. False negatives are mainly observed at the regions with overgrowth of bacteria (bacteria may produce extra fluorophores) or hyperkeratosis (keratin is strongly fluorescing). The largest effort to develop a clinical trial for autofluorescence has been explored in Canada through the Canadian Optically guided approach for Oral Lesions Surgical (COOLS) trial.^22^

### Instrumentation

**Fig. 2:**
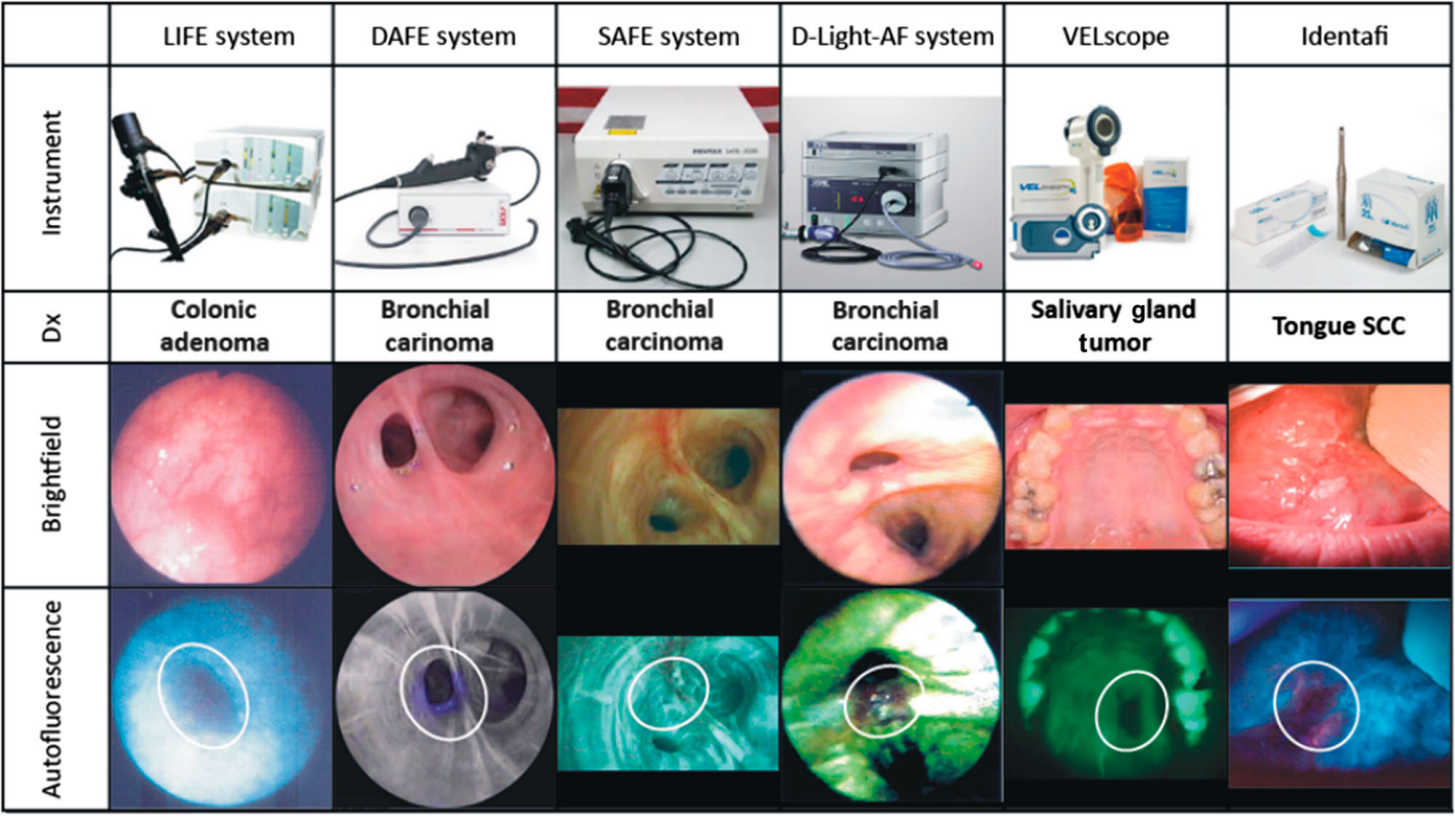
Current AFI devices to identify the neoplastic transformation and their representative images showing the tumor detections. Neoplastic tissues appear darker (due to autofluorescence loss) compared with the healthy surroundings when illuminated by AFI devices. The appearances of each instrument, diagnosis of lesions, brightfield images, and autofluorescence images are displayed. AFI, autofluorescence imaging; Dx, diagnosis; SCC, squamous cell carcinoma. Original figures can be found in refs. 23–31

Multiple AFI modalities have been proposed to detect the neoplastic transformations, such as the LIFE system (Xillix Technology, Vancouver, Canada), the DAFE system (Richard Wolf, Knittlingen, Germany), the SAFE system (Pentax, Tokyo, Japan) and D-Light-AF system (Karl Storz, Tuttlingen, Germany). These systems are not specifically designed for the head and neck region but can be applied in that area. Two AFI devices are specifically designed for inspection of the oral cavity: VELscope (LED Medical Diagnostics, Vancouver, Canada) and Identafi (DentalEZ, Lancaster, USA). The visual representations of these AFI modalities can be found in Fig. 2.^23–31^

VELscope is a non-invasive, handheld camera device for directly visualizing the alterations of tissue autofluorescence in the oral cavity.^32^ It emits blue light between 400 and 460 nm wavelengths to excite the endogenous fluorophores. After illumination, healthy tissue appears pale green when viewed through a selective long-pass filter, whereas abnormal tissue shows autofluorescence loss and appears as dark areas in contrast to the surrounding tissue. VELscope does not require special training to use and is suitable for both general or subspecialty practice. Applications include screening for abnormities, detecting neoplastic transformation, and identifying neoplasm margins.

Identafi is a multi-spectral device that incorporates three different lights: white light, violet light, and green-amber light.^33^ The white light is for conventional oral examinations, the other two lights are designed to be used sequentially to facilitate examinations. Similar to VELscope, the violet light with 405 nm wavelength utilizes the autofluorescence loss phenomenon to distinguish neoplastic tissue from normal mucosa. Similar to narrow band imaging (NBI) which will be described later, the green-amber light with 545 nm wavelength approximately matches the peaks of absorption wavelengths of hemoglobin, which may facilitate the visualization of neoangiogenesis. In addition to integrating autofluorescence and the visualization of neoangiogenic patterns into one device, the Identafi also has an advantage in its small size. The probe of Identafi resembles a dental mirror which can visualize all tissue in the oral cavity,^34^ making it considerably more flexible to handle than VELscope. However, learning to recognize neoangiogenic patterns requires a relatively steep learning curve and it, therefore, may have limited applicability to general practice.^33^

### Screening

OSCC provides an ideal model for screening and prevention because of the easy opportunity to exam the oral cavity.^35^ In 2013, Sankaranarayanan et al.^36^ published an impressive cluster randomized controlled trial with a 15 year follow-up period, which demonstrated that after three or four screening rounds with conventional oral examinations, the OSCC mortality in high-risk (tobacco and alcohol consumption) populations was significantly reduced. This study strongly confirmed the utility of conventional oral examinations in OSCC screening. The AFI devices could be used as an adjunct to conventional oral examination in OSCC or oral premalignant disease screening.

Huff et al.^37^ designed a parallel cohort study to investigate whether combining VELscope with conventional oral examination could detect more oral abnormities than conventional examination alone in a private general dentistry practice. The results suggested screening with the combination of conventional examination and VELscope yielded more mucosal abnormalities than conventional method alone (1.3% vs. 0.83%), with 83% of these being histopathology confirmed premalignant diseases. In contrast, none of abnormalities detected with conventional method alone were oral premalignacies. Similar findings from another study found adding VELscope to conventional oral examination improved the detection of oral premalignacies, which was missed by conventional examination alone.^38^ However, as previously described, the AFI devices suffer from low specificity, and this applies in screening oral lesions with VELscope.^33,39^ In an effort to improve specificity and efficacy, Bhatia et al.^40^ recently developed a decision making protocol for screening using the VELscope in general dental practice. VELscope alone showed a specificity of 54.3%, while the combination of conventional oral examination and VELscope showed a specificity of 97.9% after applying the decision making protocol.

Compared with VELscope, the Identafi is not as widely used for screening, and the studies evaluating it have been disappointing.^41^ One study specifically aimed at high-risk patients (treated previously for head and neck cancer) utilized this device for OSCC screening.^21^ The sensitivity and specificity of conventional oral examination, violet light, and green-amber light were 50% and 98%, 50% and 81%, and 0 and 86%, respectively. These unsatisfactory outcomes currently do not support its use.

### Diagnosis

The diagnostic value of AFI devices in OSCC has been widely studied. A recent meta-analysis which included 12 studies determined the accuracy of VELscope for diagnosis of OSCC and/or dysplasia.^42^ After pooling the available data, the mean sensitivity and specificity for this tool were 72.4% and 63.79%, respectively. However, the values of sensitivity ranged from 20% to 100% and specificity ranged from 15.3% to 100% according to the included studies. This may be because the autofluorescence loss phenomenon was not neoplastic specific, which would result in excessive false positives. In addition to the large standard error of pooled results, the mean sensitivity and specificity also did not support VELscope as an ideal tool for the diagnosis of oral mucosal malignant lesions at this time.

The diagnostic value of AFI devices in laryngeal cancer has also been widely investigated. Among the aforementioned AFI systems, the SAFE system (Pentax) and D-Light-AF system (Karl Storz) were the most common types applied for laryngeal cancer diagnosis. According to multiple studies, the sensitivity of SAFE system ranged between 89% and 94%, with specificity between 69% and 78%.^43–45^ For D-Light-AF system, the sensitivity ranged between 90% and 97%, and specificity ranged between 82% and 87%.^46–48^ A separate meta-analysis pooled the available data of 10 studies for AFI (combining systems) and 8 studies for white light imaging to compare diagnostic utility between the two imaging modalities. The results of sensitivity (91% AFI vs. 73% white-light imaging), specificity (84% vs. 79%), and accuracy (88% vs. 77%) of AFI were superior to white-light imaging alone.^49^ According to these studies, the AFI may be a promising tool with acceptable diagnostic value for the detection of laryngeal premalignant and cancerous lesions.

### Detecting tumor margin

As the penetrating depth of AFI illumination is relatively shallow, AFI is best suited to evaluate superficial margins. In 2006, Poh et al.^50^ first reported a case series which evaluated 20 consecutive patients with OSCC by VELscope during surgical excision. Nineteen of 20 tumors found an autofluorescence loss extending from 4 to 25 mm (mean 10.3 mm) in one or more directions beyond the clinically detectable cancer margin. Within boundaries of autofluorescence loss areas, 89% of the biopsies were pathologically confirmed cancer/dysplasia. When surgical margins were outlined at a distance of 10 mm from the boundaries of autofluorescence loss areas or clinically visible tumor (whichever was wider), biopsies showed only 1 of the 66 surgical margins was dysplastic.

A recently published retrospective study provided encouraging long-term results of VELscope-guided surgery.^51^ In patients with either early stage OSCC or high-grade lesions (severe dysplasia, carcinoma in situ), the VELscope-guided surgery group showed significant reduction in the 3-year local recurrence rate compared with the conventional surgery group, from 40.6% to 6.5% for SCC and 39.3% to 8.1% for high-grade lesions. The results also suggested that for SCC patients the VELscope-guided approach had less cervical lymph node metastasis (15.2% vs 25.0%) and rates of deaths due to disease (13.0% vs 20.3%) compared with the conventional approach, although these two differences were not statistically significant.

In 2011, the protocol for the COOLS trial was published.^22^ The COOLS trial is currently ongoing and is the first randomized, multi-center, double blind, controlled trial to validate the effectiveness of VELscope-guided surgery. This study will recruit 400 patients with SCC or high-grade dysplasia who would be randomized to either the VELscope-guided arm or the conventional white light-guided arm. The primary outcome of the study is locoregional control rate, with secondary outcomes evaluating metastasis and disease-specific survival. This study will provide level 1 clinical evidence and has implications for future practice-changing findings.

## Targeted fluorescence imaging (TFI)

### Basic theory

With the improvement in camera systems and progression in understanding cancer biology, TFI has been successfully translated to the field of surgical guidance. TFI utilizes a targeting fluorescence probe, which typically consists of a cancer targeting moiety and a conjugated fluorescent moiety to delineate neoplastic tissues and involved lymph nodes in real-time and in situ.^13^

The targeting fluorescence probe uses several mechanisms to highlight the neoplastic tissues: (1) the targeting moiety binds to the receptor of the cancer cell (e.g., epidermal growth factor receptor (EGFR), folate receptor) or is internalized into the cancer cell and then the flourescent moiety fluoresces to highlight cancer cells; (2) the quenched probe accumulated in cancer tissues is cleaved by cancer-specific enzymes (e.g., matrix metalloproteinases), resulting in de-quenched probe and a detectable fluorescence signal; (3) a combination of both ways; (4) the targeting moiety binds to the neoangiogenesis related components, such as a receptor of vascular endothelial cell surface (e.g., αvβ3 integrin) or the vascular endothelial growth factor, and then the flourescent moiety is excited and fluoresces.^52^ In addition, the high-metabolic activity of neoplastic tissues could also be targeted, similar to how 5-aminolevulinic acid (5-ALA) has been used to delineate brain gliomas.^53^

Once the targeting fluorescence probe accumulates in the neoplastic tissue, the flourescent moiety is excited by an external light source and emits photons whose signal is subsequently translated to an image by the CCD camera. The ratio of tumor signal to the surrounding signal is called tumor-to-background ratio (TBR). A TBR of at least 2 is necessary to clearly identify the tumor. NIR excitation light is less absorbed by tissue and is less interfered by autofluorescence phenomenon, which results in a higher TBR and deeper tissue penetration depth. Therefore, the ideal applied flourescent moiety is excited within the NIR spectrum.^12^

TFI can be divided into several categories according to the chemical constituent and biologic function of the targeting moiety. Among these, the targeted immune-fluorescence imaging is the most promising TFI modality in the field of head and neck cancer surgical navigation due to tissues over-expressing EGFR which can be targeted by FDA-approved EGFR antibodies (e.g., cetuximab, panitumumab). This is fortunate for targeted immune-fluorescence imaging-guided surgical navigation as the pharmacokinetic features, biodistribution, side effects, and potential toxicity of these FDA-approved antibodies are well studied.^54^ Moreover, the toxicity profile of these antibodies is usually limited to non-dose dependent events as a result of the overall required antibody dosing for targeted immune-fluorescence imaging being well below therapeutic levels.^54^

EGFR could also be targeted by nanobodies or affibodies, which are recently discovered functional antigen-binding molecules.^55,56^ A nanobody is a single-domain antibody fragment, and with a molecular weight of 15 kDa is much lighter than an antibody, making it a more efficient molecule in distribution, penetration, and clearance.^55^ An affibody is a class of even smaller (7 kDa) proteins which display binding surfaces as large as an antibody which bind with high affinity to target sites. Similar to nanobodies, affibodies also show rapid tumor targeting and clearance from body. In addition, affibodies can be designed and synthesized against antigens similar to antibodies.^56^

Even though numerous preclinical and clinical studies have built a robust landscape of targeted immune-fluorescence imaging guided cancer surgery, two fundamental challenges still exist. The first challenge is the intra-tumor phenotype heterogeneity which results from genetic and epigenetic diversity.^57^ This may have a significant impact on the sensitivity when using targeted immune-fluorescence imaging to delineate tumors as specific populations of tumor cells may downregulate the expression of cancer cell-surface antigens due to immune evasion or tumor internal coordination. The second challenge comes from the surrounding tissue’s optical properties. Scattering, absorption, and autofluorescence could cause a blurring background and low TBR, which may obscure the invasive tumor front and result in inadequate tumor resection.^57^ Several promising strategies have been proposed to solve these problems: shifting focus from cell-surface antigen to vascular related antigen to deal with tumor phenotype diversity;^58^ utilizing fluorescence differential path-length spectroscopy to quantify absorption and scattering;^59^ and applying spectral unmixing or lifetime imaging to distinguish the targeted immune-fluorescence imaging signal from autofluorescence signal.^60,61^

## Targeting fluorescence probes

Very recently, Zhang et al.^5^ specially reviewed the latest developments in cancer targeting fluorescence probes. This review is worth reading when further information is required. As previously described, the targeting fluorescence probe typically consists of a cancer targeting moiety and a conjugated fluorescent moiety. Both are of great importance in targeted immune-fluorescence imaging application and they are described separately below.

## Targeting moiety

Factors differentially expressed in tumor cells but not normal cells can be exploited to select targeted agents. These usually rely on the unique properties of cancer cells: (1) self-sufficiency in growth signals; (2) limitless replicative potential; (3) sustained angiogenesis; and (4) increased proteolytic activity resulting in tissue invasion and metastasis.^62^ A scoring system of “TArget Selection Criteria” has been described which could help to quantitatively compare potential targets.^63^ The FDA-approved antibodies are ideal targeting moiety for reasons listed above.^54^ However, for other agents, such as nanobody and affibody, further studies are still needed to confirm the in vivo characteristics of affinity, delivery, and interaction between targeting moiety and fluorescent moiety. In addition to the above targeting agents specially designed for targeted immune-fluorescence imaging, other types of agents, such as growth factors, peptides, and receptor antagonists have also been utilized for oncologic surgical navigation (Table 1).^55,64–68^

**Table 1:** Examples of currently investigated targeting fluorescence probes used for surgical guidance

Category	Tm type	Target	Probe (Tm-Fm)	Phase of development	Reference	Targeted cancer hallmarks
Immunity based	Antibody	EGFR	Cetuximab-IRDye800CW	Phases I: HNC	NCT01987375	Self-sufficiency in growth signals
		EGFR	Panitumumab-IRDye800CW	Phases I: HNC	NCT02415881	Self-sufficiency in growth signals
		VEGF	Bevacizumab-IRDye800CW	Phases II: Breast cancer	NCT02583568	Sustained angiogenesis
		TfR	TfR antibody-Alexa Fluor 680	Preclinical: HNC	Ref: 51	Limitless replicative potential
	Nanobody	EGFR	7D12-IRDye800CW	Preclinical: HNC	Ref: 42	Self-sufficiency in growth signals
	Affibody	EGFR	Affibody-IRDye800CW	Preclinical: Brain tumor	Ref: 53	Self-sufficiency in growth signals
Non-immunity based	Growth factor	EGFR	EGF-IRDye800CW	Preclinical: Brain tumor	Ref: 52	Self-sufficiency in growth signals
	Metabolized probe	Heme synthesis	5-ALA	Approved in EU for brain tumor surgery	—	Limitless replicative potential
		Folate receptor	EC17	Phases I: Breast cancer Phases I: Ovarian cancer Phases I: lung cancer	NCT01994369 NCT02000778 NCT01778920	Limitless replicative potential
	Peptide	αvβ3 integrin	AngioStamp 800	Preclinical: HNC	Ref: 53	Sustained angiogenesis
	Receptor antagonist	αvβ3 integrin	IntegriSense^TM^	Preclinical: HNC	Ref: 54	Sustained angiogenesis
	Activated probe	MMPs	MMPSense^TM^	Preclinical: HNC	Ref: 55	Tissue invasion and metastasis
		Cathepsin B	ProSense^TM^	Preclinical: HNC	Ref: 55	Tissue invasion and metastasis

*Tm*, targeting moiety, *Fm*, fluorescence moiety, *EGF*, epidermal growth factor, *EGFR*, epidermal growth factor receptor, *TfR*, transferring receptor, *HNC*, head and neck cancer, *5-ALA*, 5-aminolevulinic acid, *MMPs*, matrix metalloproteinases, *EC17*, folate conjugated to fluoresceine isothiocyanate, *VEGF*, vascular endothelial growth factor, *Ref*, reference number, *NCT*, National clinical trial number, see https://clinicaltrials.gov/

## Fluorescent moiety

Currently, there are many options for fluorescent dyes to be utilized as candidate fluorescent moiety of the probe. When selecting a suitable fluorescent moiety, several properties must be considered.^54^ The first property is interference, because only at a very low-molar ratio the fluorescent moiety could prevent interference with the antigen-binding site. The second and more important is the excitation spectrum which determines the tissue penetration and TBR. A NIR light activated fluorescent moiety is an ideal choice for the previously mentioned reasons. Besides, rapid renal clearance, low-background binding, as well as solubility and non-toxicity are also of great importance.

The fluorescent dyes currently being investigated are listed in Table 2. Among these, indocyanine green (ICG) is the only FDA-approved NIR fluorescent dye which is most commonly used for perfusion imaging and most clinical devices are tuned to this wavelength; however, it is difficult to achieve bio-conjunction with proteins.^69^ Another fluorescent dye, IRDye800CW (LICOR Biotechnology, Lincoln, US), is both NIR activable and bio-conjunctive, and is now the most widely utilized flourescent moiety in targeted immune-fluorescence imaging clinical trials even though it is not yet approved by the FDA. Fortunately, the excitation and emission wavelengths of IRDye800CW overlap with those of ICG, allowing for cost-effective and safe clinical translation by utilizing the FDA-approved NIR camera system specifically designed for ICG imaging.

**Table 2:** Currently utilized fluorescent dyes for surgical guidance

Fluorescence moiety	FDA approval (human use)	Bio-conjugation capability	Excitation wavelength/nm	Emission wavelength/nm	NIRF capability
FITC	No	Yes	495	520	No
ICG	Yes	No	807	820	Yes
Cy5.5	No	Yes	678	703	Yes
IRDye800CW	No	Yes	785	810	Yes

*FITC*, fluoresceine isothiocyanate, *ICG*, indocyanine green, *NIRF*, near-infrared fluorescence

## NIR camera system

**Fig. 3:**
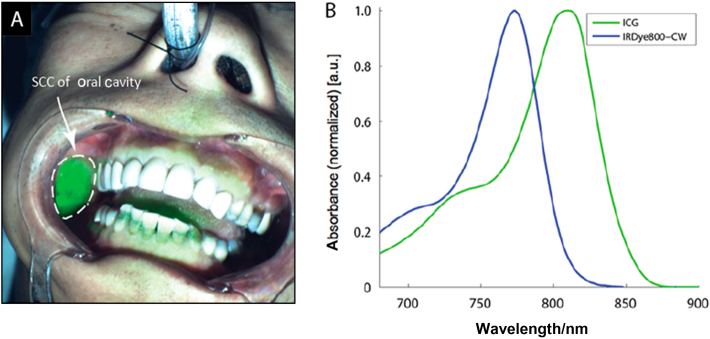
Representative TFI image and overlapping wavelength region of IRDye800CW and ICG. **a** NIR Device detecting SCC of oral cavity in Panitumumab-IRDye800CW clinical trial patient. **b** Wavelength region of IRDye800CW and ICG are overlapped, thus NIR camera system designed for ICG imaging could be utilized for TFI guided surgery.^70^ TFI, targeted fluorescence imaging; ICG, Indocyanine green; NIR: Near-infrared; SCC, squamous cell carcinoma

Targeted immune-fluorescence imaging has been relatively late to emerge because its development has been hampered by the lack of suitable NIR fluorescent dyes and dedicated NIR camera systems.^62^ The currently used NIR camera systems have been nicely reviewed elsewhere.^12,62,69^ Briefly, FDA-approved NIR camera systems for intraoperative use are in one of two formats: (1) incorporated into existing operative hardware, such as the Leica Microsystems OH5 system (Leica Microsystems, Buffalo Grove, US) and the Carl Zeiss Pentero system (Carl Zeiss Microscopy, Thornwood, US), or (2) are free standing devices specifically designed for ICG imaging, such as the SPY system (LifeCell, Branchburg, US), Fluorobeam system (Fluoptics, Grenoble, France), Photodynamic Eye system (Hamamatsu Photonics, Hamamatsu, Japan), and Luna system (Novadaq, Concord, Canada). Currently utilized NIR camera systems for surgical guidance are the latter, because ICG remains the only available NIR fluorescent dye approved by FDA for clinical use.^12^ Visual representations of TFI image overlapping wavelength region of IRDye800CW and ICG are presented in Fig. 3.^70^

## Clinical trials

Two probes designed to target the high-metabolic status of cancer cells have been translated to clinical practice. One is the precursor of the heme synthesis pathway, namely 5-ALA, the other is the folate receptor ligand conjugated to fluoresceine isothiocyanate, namely EC17. The use of 5-ALA in malignant glioma surgery^53^ and EC17 in ovarian cancer surgery^71^ were the earliest “proof-of-principle” investigations to confirm that TFI guided surgery could be used to improve surgical resections. However, these two probes are not NIR light activable probes, and therefore do not have the advantages of NIR probes described above. ICG has shown promise in sentinel lymph node mapping. However, ICG itself is not a cancer-specific agent and it utilizes the non-specific phenomenon of enhanced permeability and retention effect to delineate cancer.^12^

Targeted immune-fluorescence imaging utilizing a cancer-specific antibody conjugated to a NIR fluorescence dye is the ideal modality for fluorescence-guided cancer surgery. Currently, there are two registered phase I clinical trials in head and neck surgery which utilize the probe Cetuximab-IRDye800CW (NCT01987375) and Panitumumab-IRDye800CW (NCT02415881), both are currently enrolling at the time of this writing. Preliminary results of the NCT01987375 trial utilizing Cetuximab-IRDye800CW have been reported recently.^4,72,73^ Briefly, this study recruited patients diagnosed with HNSCC by preoperative biopsy. Twelve patients were given different doses of Cetuximab-IRDye800CW before definitive surgery and were followed up to 30 days to determine adverse events. At 3–4 days post-infusion, fluorescence imaging was performed at the beginning of surgery and intraoperatively. The samples of tumor tissue, wound bed, and normal tissue were collected and imaged ex vivo.^72^ Since this first-in-human study was initially designed to explore the safety but not interfere with standard of clinical care, all the patients underwent standard surgery protocol without fluorescence imaging navigation. In vivo results showed grade 1 adverse events attributable to the probe but no grade 2 or higher events. Intraoperative fluorescence imaging successfully differentiated tumor from normal tissue with an average TBR of 5.2 in the highest dose range.^72^ Ex vivo results obtained by assessing the resected tissue samples demonstrated that fluorescence intensity was associated with EGFR levels, but not tumor stage, tumor site, or adverse events.^72^ Using histological assessment as the gold standard to identify cancer, Cetuximab-IRDye800CW yielded an overall sensitivity of 91%, specificity of 85%, positive predictive value of 81%, and negative predictive value of 93% for 90 punch biopsy samples.^4^ When applying a ratiometric TBR threshold for determining presence of cancer by intraoperative NIR system, the threshold for tumor-to-muscle ratio was found to be 2.7, which produced a sensitivity of 90.5% and specificity of 78.6% for delineating diseased tissue, whereas tumor-to-skin ratio was found to be 1.1, which produced a higher sensitivity (92.9%) and specificity (81.0%).^73^ These promising outcomes demonstrated the low-toxicity directly and the high-accuracy indirectly; however, phase II/III trials are needed to further confirm these results.

## Narrow band imaging

### Basic theory

Neoangiogenesis is an important feature of neoplastic transformation, which may result in increased blood flow and hemoglobin proportion.^19^ Hemoglobin is a type of chromophore that only absorbs light but does not fluoresce. The absorption spectrum wavelength of hemoglobin is between 400 and 600 nm, with the peaks of absorption wavelengths of 415 and 540 nm.^74^ Thus, if a device can narrow the bandwidth of illumination light within the absorption spectrum of hemoglobin, it could help the visualization of neoangiogenic patterns inside and surrounding a target lesion (Fig. 1). Based on this hypothesis, a novel imaging modality was proposed in 2003 called narrow band imaging (NBI, Olympus Medical Systems Corporation, Tokyo, Japan).^75^

The neoangiogenic patterns under NBI examinations present as brownish or darker areas in the background of green–blue appearing normal mucosa, with scattered thick dark spots, increased microvascular density, and abnormal intraepithelial papillary capillary loops (IPCL).^76^ These neoaginogenesis-related morphological changes can be used to differentiate neoplastic tissue from normal mucosa, especially the IPCL patterns. In neoplastic lesions, the features of IPCL are dilated with a meandering course, unlike in normal mucosa. As different anatomical sites of head and neck region vary in mucosa structures, the IPCL patterns are also slightly different. These differences were detailedly summarized in a recent review article.^77^ Takano et al.^78^ developed an IPCL classification method specifically to identify the neoplastic transformation of oral mucosa when applying NBI. In this classification, IPCL patterns are divided into 4 types: (1) type I or normal: IPCL are perpendicular to the mucosal surface, and loops appear to have both waved arms together; (2) type II or dilation, IPCL appear a similar shape to type I but with notably increased caliber; (3) type III or elongation: IPCL are elongated or appear in tangled lines, and often accompanied with dilation; (4) type IV or destruction: IPCL appear as large vessels with no terminal loops. The destruction of IPCL structure is due to progressive dilation and elongation. Among these IPCL patterns, type III and IV are indicative of neoplastic lesions, although some non-neoplastic lesions, such as leukoplakia, can also present with type III IPCL pattern.^78^

Although, widely available for many years and widely available on commercial systems, the technique has not gained significant traction clinically in the United States. This is perhaps because it has several limitations. First, characterizing the IPCL patterns is subjective and requires a relatively long period to master, so it may result in low-diagnostic accuracy with unnecessary biopsies in the early phase of the learning curve.^79^ The second limitation is visualization of neoaginogenesis architecture may be affected by varying tissue characteristics, such as the level of keratinization, epithelium thickness/stratification, and the presence of lymphoid tissue.^80^ NBI is designed for recognizing neoangiogenesis patterns, so scenarios with modified microvascularity, such as previous radiation or surgery, inflammation and vascular lesions, can lead to false positive results.^81^

## Instrumentation

The NBI system is an endoscopic technology that is widely available on most commercial flexible endoscopy devices and allows the user to switch between white light mode and NBI mode. NBI mode simultaneously emits both blue light (400–430 nm, centered at 415 nm) and green light (525–555 nm, centered at 540 nm) that approximately match the peaks of absorption wavelengths of hemoglobin to enhance the visualization of microvascular patterns.^33^ The blue light has shorter wavelength with shallow penetration to highlight the superficial vessels, while the green light with longer wavelength penetrates deeper to illuminate underlying vessels.^33^ Switching between the white mode and NBI mode can be easily achieved with the press of a button, and this process can be repeated several times during one examination.^76^ Moreover, improvement of microvascular pattern visualization could be achieved by combining the NBI system with magnifying endoscopy and high-definition camera.^33,76^

## Clinical trials

Multiple studies have reported that NBI could detect early HNSCC efficiently and more frequently than conventional white-light imaging in high-risk population.^82–84^ Recently, Nakanishi et al.^85^ published a large study with the objective of detecting pharyngeal cancer in the general population undergoing upper gastrointestinal endoscopy using NBI. In the screening group with 8872 participants, 10 patients were detected with pathologically confirmed superficial or early stage pharyngeal cancer. Although, the study did not report on the diagnostic accuracy and missed diagnosis rate of NBI, this study highlighted the role of NBI in routine HNSCC screening.

First used in gastroenterology, NBI has been extensively utilized for diagnosing HNSCC with very encouraging results. In 2013, Li et al.^86^ performed a meta-analysis of 21 studies utilizing NBI in the evaluation of mucosal and sub-mucosal malignant lesions in the head and neck region. The overall sensitivity (90% vs. 62%), specificity (97% vs. 85%), and accuracy (98% vs. 89%) of NBI were superior to white-light imaging examination. Subgroup analysis based on anatomical sites (nasopharynx, oral cavity/oropharynx, and larynx) also achieved similar results. Additional recent studies have been published in the otolaryngology literature, suggesting the diagnostic value offered by NBI in defining head and neck lesions.^87–89^

There is some controversy over the definition of a positive lesion. Most studies regard the “well-demarcated brownish area with thick dark spots and/or winding vessels” as the positive lesions using NBI.^79^ However, Lin et al.^80^ argued that the prevalence of brownish spots, which have a higher frequency of occurrence in the floor of mouth, hypopharynx, and epiglottis, is not consistent across all areas of the head and neck region. Thus the “brownish area” is not a universally accepted positive finding in the head and neck and further studies to establish a standard for “positive” need to occur.

Recently, there have been several retrospective non-randomized controlled studies to explore the effect of intraoperative NBI examination on the incidence of positive superficial surgical margins in HNSCC.^90–92^ These studies drew the resection lines in accordance with (but not exceeding) the NBI defined positive areas. Similar to AFI, NBI is only useful on superficial mucosa so all the studies analyzed only the superficial surgical margins. Garofolo et al.^90^ examined 82 patients with Tis-T1a glottic cancer treated with transoral laser microsurgery with intraoperative NBI margin evaluation. The definitive histology showed the rate of positive superficial margins was significantly lower than control group (3.6% vs. 23.7%). Vicini et al.^91^ evaluated 58 patients with confirmed HNSCC who underwent transoral robotic surgery procedures. Patients were separated into receiving intraoperative NBI evaluation or standard white-light imaging evaluation. Frozen section analysis of surgical margins revealed a significantly lower rate of positive superficial margins in the NBI group compared with the white-light imaging group (12.1% vs. 42.1%). Tirelli et al.^92^ evaluated the superficial surgical margins in oral and oropharyngeal tumors with intraoperative frozen section analysis and definitive histology and compared with a historical cohort. In contrast with Vicini’s study, Tirelli’s study found no significant difference in frozen section margins between NBI group and white-light imaging group, but the definitive histology observed a significant reduction in the rate of positive superficial margins in NBI group (11.5% vs. 36.4%).^92^ Interestingly, this study also calculated the enlargement of the resection based on NBI measurement and found that the resection was performed at a mean distance of 2.5 cm from the macroscopic edge of the tumor. Based on the above results, the authors stated that these findings might challenge the dogma that maintaining a resection margin of 1.5 cm represents the best compromise between complete resection and tissue reservation.^92^

## High-resolution microendoscopy

### Basic theory

HRME is a cost-effective, non-invasive and probe-based HRME, which is performed by placing the flexible fiber-optic probe in direct contact with the suspicious mucosal surface which is preferentially stained with a fluorescent contrast agent. Illumination then occurs using a light-emitting diode (LED) transmitted through the fiber-optic bundle which excites endogenous or superficially applied fluorophores. Simultaneous with the LED illumination, the emitted light is collected by the probe, with each optical fiber serving as an individual pixel of the image, and then directed into a CCD camera. The camera is connected to a computer to present videos and images which typically show bright nuclei on a dark background.^93^ With sub-cellular resolution (4.4 μm, ×1 000 magnification) imaging capability and a frame rate of 10–15 fps, the HRME can provide microscopic images of the cellular architecture of selected tissue in situ and in real-time, thus achieving the so-called “optical biopsy”.^93,94^ The criterion for distinguishing neoplastic tissue from benign epithelium using HRME is based on histological features, including nuclear size, crowding, nuclear-to-cytoplasm ratio (N/C ratio), and overall cellular pleomorphism.^6^ “Normal tissue” diagnosed by HRME refers to images where cell nuclei appear as bright discrete dots evenly distributed throughout the field-of-view, whereas the “neoplastic tissue” typically contains images with crowded and enlarged cell nuclei that are chaotically arranged.

In contrast to other high-resolution imaging modalities (e.g., optical coherence tomography, confocal laser endomicroscopy), the HRME device has unique advantages which may enable its widespread clinical application. First, the technique has a sensitivity and specificity of 98% and 92%, respectively, for the ex vivo detection of HNSCC.^6^ Second, the optical imaging system of HRME is relatively concise as it requires no scanning mirrors, complex light sources, or other moving parts. This results in a simple and portable device and significantly decreases the overall cost for production and maintenance.^93^ Finally, because of the similarity with conventional H&E histopathology, the HRME system requires minimal training time for clinicians to identify dysplastic and neoplastic lesions. Post-training accuracy values are similar between inexperienced HRME clinicians and experts, which suggests a high degree of inter-rater reliability when interpreting HRME images.^6^

## Instrumentation

Unfortunately, the complete HRME package is not commercially available at present. However, it can be built using commercially available components, including the fiber-optic bundle, lens, filter, mirror, LED, optomechanical positioning component, and a laptop or desktop computer.^95^ After assembly, the HRME device can be functionally divided into three parts: a thin-flexible fiber-optic probe, a combined light source and camera, and a laptop or tablet based processor.^1^ The whole system is portable as it can be packaged into a box and the electrical components powered by a battery pack or USB ports of the host computer.^95^ The spatial resolution of HRME is affected by inter-fiber spacing, usually at 4 μm. Additional magnification can be provided by a micro-lens or graded-index lens bonded to the distal tip.^95^ The field-of-view of HRME initially depends on the diameter of the active area of the fiber bundle (from 330 to 1400 μm, usually 720 μm). The smaller bundles can be inserted through the lumen of a narrow gauge hypodermic needle and are significantly more flexible than the larger fibers.^95^ The degree of demagnification is proportional to the increase in spatial resolution and it correspondingly decreases the field-of-view.

Several different fluorescent contrast agents to identify the nuclear material have been studied for HRME imaging, including benzoporphyrin-derivative monoacid ring A,^96^ fluoroscein,^97^ and proflavine.^6^ The proflavine, which is the most commonly used contrast agent for HRME, is an acridine-derived dye that reversibly binds to DNA and stains cell nuclei with a peak excitation and emission wavelength of 445 nm and 515 nm, respectively.^95^ Although, previous studies reported no adverse effects when applied for gastrointestinal fluorescence imaging,^98^ proflavine is yet not FDA approved for in vivo clinical use as a topical contrast agent (a result of lacking long-term study of mutagenic effects in humans).^93^ Another problem with proflavine is its high affinity for keratin. Hence, it can be challenging to interpret images of proflavin-enhanced HRME in the setting of heavily keratinized mucosa, such as the hard palate and gingiva mucosa.^6^

The innately simple design of HRME does not allow optical sectioning, and the 455 nm excitation wavelength of proflavine may only penetrate the epithelium to a depth corresponding to a few cell layers (~50 μm). Therefore, HRME imaging is limited to the superficial mucosa and is unable to inspect submucosal tumors or submucosal tumor spread.^6,93^ This limitation may be addressed by changing the fluorophore to one excited by a light with deeper tissue penetration (i.e., NIR wavelength), or submucosal delivery of the fiber-optic probe by inserting it into a 16-gauge needle which penetrates into deeper layers of the epithelium. The field-of-view of HRME is also inherently limited by the diameter of the bundle.^93^ A small bundle can only interrogate a limited area of tissue, which introduces an opportunity to miss occult disease due to sampling error. This problem may be addressed by algorithms for real-time video mosaicing that can effectively increase the acquired image size.^99^

## Clinical trials

Current clinical applications of HRME in the head and neck are limited to distinguishing neoplastic tissues from normal mucosa in a diagnostic setting. Screening for lesions is not practical for HRME due to the restricted field-of-view. The HRME device might be a promising tool in real-time assessment of surgical margins; however, there have been no studies investigating this capability, possibly also as a result of the narrow field-of-view.

Several studies have been published on ex vivo study investigating the diagnostic value of HRME for identifying HNSCC tissue samples. Vila et al.^6^ involved 38 patients who had primary HNSCC diagnosed by prior biopsy. After surgical resection, the resected tissue samples were immediately stained with proflavine and imaged by HRME at multiple regions of interest (ROI), including suspected tumor, adjacent benign-appearing mucosa, and transition areas. After imaging, the ROI samples were correlated with standard histopathology. After a brief training with representative HRME images labeled with pathological diagnosis, seven head and neck pathologists without previous HRME experience were asked to blindly interpret the HRME images. The results demonstrated that the sensitivity, specificity, and kappa statistic for inter-rater reliability was 0.98 (95% CI, 0.97–1.00), 0.91 (95% CI, 0.85–0.97), and 0.84 (95% CI, 0.77–0.91), respectively. In 2013, they enlarged the sample size and published another study with the same methods described above but included HRME video format.^100^ Similar to the previous study, the sensitivity, specificity, and inter-rater reliability provided by still images was excellent (98%, 92%, and 84%, respectively). However, the sensitivity, specificity, and inter-rater reliability provided by videos decreased to 84%, 68%, and 0.47%, respectively. Taken together, it can be concluded that HRME permits accurate discrimination of benign and malignant mucosa ex vivo, and may have the potential to be applied in vivo.

Based on the study design of the two ex vivo studies, Miles et al.^101^ designed a prospective, phase I trial of in vivo HRME imaging. This trial enrolled 38 primary HNSCC patients and provided still images to 11 head and neck pathologists for interpretation. As an in vivo study, the image data of HRME was collected by directly placing the probe on the mucosal surface of oral cavity, oropharynx, and larynx. Outcomes of this study were similar to the ex vivo studies: the mean accuracy in identifying neoplastic or benign mucosa was 0.951 (95% CI, 0.94–0.96); sensitivity, specificity and inter-rater reliability were 0.96 (95% CI, 0.94–0.99), 0.95 (95% CI, 0.90–0.99), and 0.84 (95% CI, 0.78–0.84), respectively.

Further studies have demonstrated that HRME can be combined with wide-field AFI devices. Pierce et al.^102^ developed a multimodal optical imaging system which combined AFI and HRME to evaluate oral lesions at both macroscopic and microscopic levels. After interpreting 100 OSCC ROI, this system correctly classified 98% of pathologically confirmed normal ROI, and 95% of ROI graded as neoplastic. When stratified by p63 status, HRME, AFI or the combined system could correctly classify 73%, 67%, or 87% respectively, of pathologically confirmed mild dysplasia (often considered the most difficult to classify by any imaging modality). This study introduces the possibility of using combined optical imaging devices for real-time, in vivo delineation of pre-cancerous mucosa with molecular damage.

## Algorithms

Diagnostic algorithms have been developed for quantitatively analyzing HRME images and determining N/C ratio.^102–104^ Higher N/C ratios result from enlarged or crowded nuclei, which allow the N/C ratio to be used to classify ROI as non-neoplastic or neoplastic. The algorithm used proflavine-enhanced HRME images to reveal cell nuclei as discrete bright dots on a dark background, allowing a binary image to be constructed. Pixels corresponding to nuclei area were counted and divided by the total number of pixels in the ROI, yielding the N/C ratio.^102,103^ In 2012, Pierce et al.^102^ published a study investigating the accuracy of in vivo HRME imaging for detection of oral neoplasia using the N/C ratio-calculating algorithm. This study included 30 patients with clinically visible oral lesions and obtained 100 images of ROI. Of the 100 ROI, 45 were non-neoplastic and 55 were neoplastic (including mild/moderate/severe dysplasia, and cancer) confirmed by histopathology. With a threshold value of 0.142 N/C ratio, the sensitivity and specificity was 84% and 71%, respectively. Representative still images are manually selected by clinicians and can be time-consuming, so Ishijima et al.^104^ developed a novel automated frame selection algorithm for HRME video sequences. They tested their algorithm using the same data set and protocol as the study of Pierce et al., also using the N/C ratio to identify neoplastic tissue. With an N/C ratio threshold value of 0.25, the algorithm correctly classified these ROI with 71% sensitivity and 80% specificity for manually selected frames, as well as 69% sensitivity and 76% specificity for automatically selected frames. Although the accuracy outcomes of this study are not ideal and improvements are clearly required, the combination of these two algorithms may 1 day allow fully automated diagnosis with HRME in real time.

## Raman spectroscopy (RS)

### Basic theory

RS is a vibrational spectroscopic technique that can detect the variations of chemical components and capture the ‘molecular fingerprint’ of the tissue. In 1928, C.V. Raman discovered that vibrations of intramolecular bonds caused light to scatter as a result of absorption or release of energy, which was named Raman scattering. This scattering could be captured and measured, forming a spectrum. A Raman spectrum contains a series of specific and characteristic peaks or bands assigned to a corresponding molecular structure and biochemical composition within tissue.^105^ RS shows several advantages in comparison to other spectroscopic methods:^8,106^ (1) in contrast to infrared spectroscopy, water absorption does not disturb the measurement; (2) using excitation at 1 064nm by means of an Nd: YAG-laser virtually eliminates fluorescence; (3) the typically high signal-to-noise ratio of the Raman spectrum allows the use of chemometric methods of measurement; (4) due to the smaller diameter of the laser beam, fewer sample volumes are required for spectroscopic analysis. There is an abundance of information in the spectra, and the data cannot be analyzed simply by observation and comparison, so chemometric methods play essential roles in the analysis of RS and improve sensitivity.^106^ Multiple analytic methods exist (principal component analysis, linear discriminant analysis, support vector machines, neural network analysis, etc.) to understand and use the data with the goal of developing models to help with screening, diagnosis, and treatment evaluation.

## Clinical Studies

RS has been successfully applied to various organ systems, including diagnoses of premalignant and malignant lesions in stomach,^107^ skin,^108,109^ colon,^110,111^ esophagus,^112,113^ bladder,^114,115^ and the prostate gland.^116,117^ Very recently, Santos et al.^118^ reviewed the state of art of in vivo and ex vivo oncological applications of RS. Besides the applications in each cancer types, this review also concluded the current chances and challenges of the instrumentation and transferability of RS.

RS was first applied in the head and neck by Stone et al.^119^ in 2000 to analyze laryngeal mucosa ex vivo using biopsy specimens from 15 patients. Each biopsy was divided in two and underwent either histopathologic analysis or RS for 30 s. Reference spectra were generated from seven patients with histopathologically normal mucosa. Multivariate statistical analysis of the data was carried out to evaluate and maximize the differences in the spectra. In the study, RS demonstrated a specificity of 90% and sensitivity of 92% for diagnosing invasive cancer. Researchers analyzed biopsy specimens from the vocal cords of 20 patients using RS recorded over 1–30 s. Multivariate analysis was used to determine prediction sensitivities of 89%, 69%, and 88%, and specificities of 86%, 94%, and 94% for normal tissue, carcinoma, and papilloma, respectively.^120^ Guze et al.^121^ utilized RS to identify spectral differences between normal and malignant squamous cells in oral mucosa. Multivariate analysis showed that premalignant and malignant lesions could be predicted with 100% sensitivity and 77% specificity. In order to evaluate RS in detecting premalignant conditions, Singh et al.^122^ obtained the Raman spectra from premalignant patches, normal, and cancerous sites in oral mucosal samples. They were able to differentiate the premalignant conditions based on the differences between the spectra of biopsies.

Fourier-transform filters interfering fluorescence signals which allows for improved detection of weak Raman signals. Oliveira et al.^123^ showed that an algorithm based on principal component analysis was able to separate the samples into a normal group and carcinoma group. Li et al. established diagnostic models by using the Raman spectra generated by Fourier-transform near-infrared Raman spectrometer. The diagnostic models performed well in discriminating normal mucosa from leukoplakia and SCC. However, the normal versus the low-grade leukoplakia as well as the high-grade leukoplakia versus SCC could not be accurately classified because of the high similarity of the Raman spectra of the biopsies in these two compared groups.^106^ Yan et al.^124^ used RS at 785 nm to scan the tissue samples of pleomorphic adenoma, Warthin’s tumor, and normal tissues of parotid gland, and then applied support vector machines to establish a diagnostic model. The results showed that RS can detect the biochemical variations between the normal tissues and tumors, and the overall accuracy was better than 95% in all the paired groups. The same groups later employed surface-enhanced RS to analyze biochemical changes in the blood serum between the parotid gland tumor groups and normal control group, and the results showed that nucleic acids and proteins increased in the spectra of the parotid gland tumor serums, allowing for prediction of the tumor group with high accuracy (84.1%–88.3%), sensitivity (82.2%–97.4%), and specificity (73.7%–86.7%).^125^

RS also had potential in tumor margin discrimination. The above cited articles mainly aimed at discriminating different stages of mucosa neoplasia, and the investigated mucosa is limited to superficial layer. However, when it comes to surgery, the tumor-surrounding tissues are not only the superficial mucosa, but also subepithelial tissues, such as the connective tissue, muscle, adipose and so forth. In 2015, Cals et al.^126^ investigated the application of RS in discriminating between OSCC and individual surrounding tissues. The linear discriminant analysis models could distinguish OSCC from adipose tissue, nerve, muscle, gland, connective tissue, and squamous epithelium in 100%, 100%, 97%, 94%, 93%, and 75% of the cases, respectively. Then in 2016, the same team developed in vitro RS-based tissue classification models for distinguishing OSCC from subepithelial non-cancerous tissue. By utilizing the developed method, RS showed an accuracy of 91%.^127^ Besides the spectral differences between OSCC and surrounding tissues, recent studies have showed that the water concentration determined by RS might enable locating the OSCC border. In 2015, Barroso et al.^128^ conducted a pilot study and found that using water concentration as the discriminating factor, RS could discriminate tumor from surrounding tissue with a sensitivity of 99% and a specificity of 92%. In 2016, they found a more interesting phenomenon that the water concentration and its corresponding concentration heterogeneity were significantly different between the regions: the water concentration in tumor is 76% ± 8%, in the inadequate margin (0–5 mm) it is 59% ± 24%, and in the adequate margin (>5 mm) it is 54% ± 24%.^129^ Actually, RS is very suitable for detecting the water concentration in tissue because the whole process is rapid, quantitative, and objective. Utilizing the water concentration as the discriminating factor, RS may be implemented for rapid intraoperative assessment of margin state.

Although, RS is a sensitive diagnostic technique, it has not been developed for commercial applications. The development of optical fibers allows the sampling location to be independent of the spectrometer and could play an important role in laser transduction and signal collection in future RS in vivo clinical applications. Methods for data mining in RS research, while suitable for classification of different tissues, need improvement in efficiency to be used for clinical applications. As new chemometric methods are developed, it is important to have collaboration between the clinicians and chemical scientists to establish more effective and efficient tools for the medical applications.

## Future directions

Although, the field of in vivo optical imaging has been developing for decades, there is still significant opportunity. Many instruments, such as the Raman spectrometer, are not commercially available and not portable or are too large for routine clinical use. With support from commercial industries, some instruments such as VELscope in AFI and SPY in TFI, have entered into the operating room in trial settings. However, more rigorous clinical trials need to be performed to demonstrate clinical benefit when these devices are applied, rather than just diagnostic accuracy.

Combining new technologies with those which already exist, such as NBI with the surgical robot, or using existing surgical microscopes for immunofluorescence, is a way to implement these technologies in an efficient and more cost-effective manner. Continued merging of existing and new technology is necessary and encouraged, especially as the field of transoral robotic surgery where the lack of tactile feedback and low-light environment provides an optimal opportunity for these technologies. Finally, routine collaboration between basic scientists, physicists, radiologists, pathologists, surgeons, and industry is necessary to identify methodologies to demonstrate the potential improvement in care associated with these techniques.

## References

[1] Shield KD (2017). The global incidence of lip, oral cavity, and pharyngeal cancers by subsite in 2012. Cancer J. Clin..

[2] Hashim D (2016). The role of oral hygiene in head and neck cancer: results from International Head and Neck Cancer Epidemiology (INHANCE) consortium. Ann. Oncol..

[3] Krishna Rao SV, Mejia G, Roberts-Thomson K, Logan R (2013). Epidemiology of oral cancer in Asia in the past decade--an update (2000-2012). Asian Pac. J. Cancer Prev..

[4] Warram JM (2016). Fluorescence imaging to localize head and neck squamous cell carcinoma for enhanced pathological assessment. J. Pathol. Clin. Res..

[5] Zhang RR (2017). Beyond the margins: real-time detection of cancer using targeted fluorophores. Nat. Rev. Clin. Oncol..

[6] Vila PM (2012). Discrimination of benign and neoplastic mucosa with a high-resolution microendoscope (HRME) in head and neck cancer. Ann. Surg. Oncol..

[7] Vu A, Farah CS (2016). Narrow band imaging: clinical applications in oral and oropharyngeal cancer. Oral Dis..

[8] Harris AT (2010). Raman spectroscopy in head and neck cancer. Head Neck Oncol..

[9] Davies K (2015). Point of care optical diagnostic technologies for the detection of oral and oropharyngeal squamous cell carcinoma. Surgeon.

[10] Green B, Cobb AR, Brennan PA, Hopper C (2014). Optical diagnostic techniques for use in lesions of the head and neck: review of the latest developments. Br. J. Oral. Maxillofac. Surg..

[11] Green B, Tsiroyannis C, Brennan PA (2016). Optical diagnostic systems for assessing head and neck lesions. Oral Dis..

[12] Rosenthal EL, Warram JM, Bland KI, Zinn KR (2015). The status of contemporary image-guided modalities in oncologic surgery. Ann. Surg..

[13] de Boer E (2015). Optical innovations in surgery. Br. J. Surg..

[14] Keereweer S (2013). Optical image-guided cancer surgery: challenges and limitations. Clin. Cancer Res..

[15] Yang Q (2017). Rational design of molecular fluorophores for biological imaging in the NIR-II Window. Adv. Mater.

[16] Sturm MB, Wang TD (2015). Emerging optical methods for surveillance of Barrett’s oesophagus. [Review]. Gut.

[17] de Veld DC (2005). Autofluorescence and Raman microspectroscopy of tissue sections of oral lesions. Lasers Med. Sci..

[18] Lane PM (2006). Simple device for the direct visualization of oral-cavity tissue fluorescence. J. Biomed. Opt..

[19] Messadi DV (2013). Diagnostic aids for detection of oral precancerous conditions. Int. J. Oral. Sci..

[20] Mercadante V, Paderni C, Campisi G (2012). Novel non-invasive adjunctive techniques for early oral cancer diagnosis and oral lesions examination. Curr. Pharm. Des..

[21] Sweeny L (2011). Assessment of tissue autofluorescence and reflectance for oral cavity cancer screening. Otolaryngol. Head Neck Surg..

[22] Poh CF (2011). Canadian Optically-guided approach for Oral Lesions Surgical (COOLS) trial: study protocol for a randomized controlled trial. BMC Cancer.

[23] LIFE system introduction. Available at http://www.medwow.com/med/video-endoscopy/xillix/onco-life/45863.model-spec.

[24] Ogihara T (1999). Clinical experience using a real time autofluorescence endoscopy system in the gastrointestinal tract. Diagn. Ther. Endosc..

[25] DAFE system introduction. Available at http://www.richard-wolf.com/broschueren/Pneumonology/G_646_Dafe_Diagnostische_Auto_Fluoreszenz_en_08.pdf.

[26] SAFE system introduction. Available at http://pdf.medicalexpo.com/pdf/pentax/safe-3000/70880-86807.html.

[27] D-Light-AF system introduction. Available at https://www.karlstorz.com/au/en/fi.htm.

[28] Hausinger K, Stanzel F, Huber RM, Pichler J, Stepp H (1999). Autofluorescence detection of bronchial tumors with the D-Light/AF. Diagn. Ther. Endosc..

[29] VELscope introduction. Available at https://www.pattersondental.com/Supplies/ProductFamilyDetails/PIF_614957.

[30] Visual representation of VELscope identified neoplastic tissue. Available at http://www.identalcentre.com/services/oral-cancer-exams/velscope-oral-cancer-exam/.

[31] Identafi introduction. Available at http://www.dentalez.com/products/stardental/identafi.

[32] Farah CS, McIntosh L, Georgiou A, McCullough MJ (2012). Efficacy of tissue autofluorescence imaging (VELScope) in the visualization of oral mucosal lesions. Head Neck.

[33] Bhatia N, Lalla Y, Vu AN, Farah CS (2013). Advances in optical adjunctive AIDS for visualisation and detection of oral malignant and potentially malignant lesions. Int. J. Dent..

[34] Roblyer D (2009). Objective detection and delineation of oral neoplasia using autofluorescence imaging. Cancer Prev. Res..

[35] Iyer S, Thankappan K, Balasubramanian D (2016). Early detection of oral cancers: current status and future prospects. Curr. Opin. Otolaryngol. Head Neck Surg..

[36] Sankaranarayanan R (2013). Long term effect of visual screening on oral cancer incidence and mortality in a randomized trial in Kerala, India. Oral Oncol..

[37] Huff K, Stark PC, Solomon LW (2009). Sensitivity of direct tissue fluorescence visualization in screening for oral premalignant lesions in general practice. Gen. Dent..

[38] Truelove EL (2011). Narrow band (light) imaging of oral mucosa in routine dental patients. Part I: assessment of value in detection of mucosal changes. Gen. Dent..

[39] McNamara KK, Martin BD, Evans EW, Kalmar JR (2012). The role of direct visual fluorescent examination (VELscope) in routine screening for potentially malignant oral mucosal lesions. Oral Surg. Oral Med Oral Pathol. Oral Radiol..

[40] Bhatia N, Matias MA, Farah CS (2014). Assessment of a decision making protocol to improve the efficacy of VELscope in general dental practice: a prospective evaluation. Oral Oncol..

[41] Lalla Y, Matias M, Farah CS (2015). Oral mucosal disease in an Australian urban Indigenous community using autofluorescence imaging and reflectance spectroscopy. Aust. Dent. J..

[42] Giovannacci I, Vescovi P, Manfredi M, Meleti M (2016). Non-invasive visual tools for diagnosis of oral cancer and dysplasia: a systematic review. Med. Oral Patol. Oral Cir. Bucal.

[43] Baletic N, Petrovic Z, Pendjer I, Malicevic H (2004). Autofluorescent diagnostics in laryngeal pathology. Eur. Arch. Otorhinolaryngol..

[44] Baletic N, Malicevic H, Petrovic Z, Marinkovic-Eric J, Peric A (2010). Advantages and limitations of the autofluorescent diagnostics of the laryngeal cancer and precancerosis. Eur. Arch. Otorhinolaryngol..

[45] Caffier PP (2013). A comparison of white light laryngostroboscopy versus autofluorescence endoscopy in the evaluation of vocal fold pathology. Laryngoscope.

[46] Malzahn K, Dreyer T, Glanz H, Arens C (2002). Autofluorescence endoscopy in the diagnosis of early laryngeal cancer and its precursor lesions. Laryngoscope.

[47] Arens C, Dreyer T, Glanz H, Malzahn K (2004). Indirect autofluorescence laryngoscopy in the diagnosis of laryngeal cancer and its precursor lesions. Eur. Arch. Otorhinolaryngol..

[48] Arens C (2007). Indirect fluorescence laryngoscopy in the diagnosis of precancerous and cancerous laryngeal lesions. Eur. Arch. Otorhinolaryngol..

[49] Kraft M, Betz CS, Leunig A, Arens C (2011). Value of fluorescence endoscopy for the early diagnosis of laryngeal cancer and its precursor lesions. Head Neck.

[50] Poh CF (2006). Fluorescence visualization detection of field alterations in tumor margins of oral cancer patients. Clin. Cancer Res..

[51] Poh CF (2016). Fluorescence visualization-guided surgery for early-stage oral cancer. JAMA Otolaryngol. Head Neck Surg..

[52] Keereweer S (2012). Image-guided surgery in head and neck cancer: current practice and future directions of optical imaging. Head Neck.

[53] Stummer W (2006). Fluorescence-guided surgery with 5-aminolevulinic acid for resection of malignant glioma: a randomised controlled multicentre phase III trial. Lancet Oncol..

[54] Warram JM (2014). Antibody-based imaging strategies for cancer. Cancer Metastas-. Rev..

[55] van Driel PB (2014). Intraoperative fluorescence delineation of head and neck cancer with a fluorescent anti-epidermal growth factor receptor nanobody. Int. J. Cancer.

[56] Bai M, Bornhop DJ (2012). Recent advances in receptor-targeted fluorescent probes for in vivo cancer imaging. Curr. Med. Chem..

[57] Keereweer S, Van Driel PB, Robinson DJ, Lowik CW (2014). Shifting focus in optical image-guided cancer therapy. Mol. Imaging Biol..

[58] Radu A (2010). Expression of follicle-stimulating hormone receptor in tumor blood vessels. N. Engl. J. Med..

[59] Kruijt B (2009). In vivo quantification of chromophore concentration using fluorescence differential path length spectroscopy. J. Biomed. Opt..

[60] Zimmermann T (2005). Spectral imaging and linear unmixing in light microscopy. Adv. Biochem. Eng. Biotechnol..

[61] Sun Y (2010). Fluorescence lifetime imaging microscopy for brain tumor image-guided surgery. J. Biomed. Opt..

[62] Keereweer S (2011). Optical image-guided surgery--where do we stand?. Mol. Imaging Biol..

[63] van Oosten M, Crane LM, Bart J, van Leeuwen FW, van Dam GM (2011). Selecting potential targetable biomarkers for imaging purposes in colorectal cancer using TArget Selection Criteria (TASC): a novel target identification tool. Transl. Oncol..

[64] Shan L (2008). Visualizing head and neck tumors in vivo using near-infrared fluorescent transferrin conjugate. Mol. Imaging.

[65] Gong H, Kovar JL, Cheung L, Rosenthal EL, Olive DM (2014). A comparative study of affibody, panitumumab, and EGF for near-infrared fluorescence imaging of EGFR- and EGFRvIII-expressing tumors. Cancer Biol. Ther..

[66] Atallah I (2016). Near-infrared fluorescence imaging-guided surgery improves recurrence-free survival rate in novel orthotopic animal model of head and neck squamous cell carcinoma. Head Neck.

[67] Keereweer S (2012). Targeting integrins and enhanced permeability and retention (EPR) effect for optical imaging of oral cancer. J. Surg. Oncol..

[68] Keereweer S (2011). Detection of oral squamous cell carcinoma and cervical lymph node metastasis using activatable near-infrared fluorescence agents. Arch. Otolaryngol. Head Neck Surg..

[69] Schaafsma BE (2011). The clinical use of indocyanine green as a near-infrared fluorescent contrast agent for image-guided oncologic surgery. J. Surg. Oncol..

[70] Taruttis A, Morscher S, Burton NC, Razansky D, Ntziachristos V (2012). Fast multispectral optoacoustic tomography (MSOT) for dynamic imaging of pharmacokinetics and biodistribution in multiple organs. PLoS ONE.

[71] van Dam GM (2011). Intraoperative tumor-specific fluorescence imaging in ovarian cancer by folate receptor-alpha targeting: first in-human results. Nat. Med..

[72] Rosenthal EL (2015). Safety and tumor specificity of cetuximab-IRDye800 for surgical navigation in head and neck cancer. Clin. Cancer Res..

[73] Warram JM (2015). A ratiometric threshold for determining presence of cancer during fluorescence-guided surgery. J. Surg. Oncol..

[74] Subhash N (2006). Oral cancer detection using diffuse reflectance spectral ratio R540/R575 of oxygenated hemoglobin bands. J. Biomed. Opt..

[75] Gono K (2003). Endoscopic observation of tissue by narrowband illumination. Opt. Rev..

[76] Piazza C, Del Bon F, Peretti G, Nicolai P (2012). Narrow band imaging in endoscopic evaluation of the larynx. Curr. Opin. Otolaryngol. Head Neck Surg..

[77] Ni XG, Wang GQ (2016). The role of narrow band imaging in head and neck cancers. Curr. Oncol. Rep..

[78] Takano JH (2010). Detecting early oral cancer: narrowband imaging system observation of the oral mucosa microvasculature. Int. J. Oral. Maxillofac. Surg..

[79] Piazza C (2011). Narrow band imaging and high definition television in the endoscopic evaluation of upper aero-digestive tract cancer. Acta Otorhinolaryngol..

[80] Lin YC, Wang WH, Lee KF, Tsai WC, Weng HH (2012). Value of narrow band imaging endoscopy in early mucosal head and neck cancer. Head Neck.

[81] Chu PY, Tsai TL, Tai SK, Chang SY (2012). Effectiveness of narrow band imaging in patients with oral squamous cell carcinoma after treatment. Head Neck.

[82] Saito M (2013). Pharyngeal cancer surveillance using narrow band imaging during conventional upper gastrointestinal endoscopy. Digestion.

[83] Muto M (2010). Early detection of superficial squamous cell carcinoma in the head and neck region and esophagus by narrow band imaging: a multicenter randomized controlled trial. J. Clin. Oncol..

[84] Nonaka S, Saito Y, Oda I, Kozu T, Saito D (2010). Narrow-band imaging endoscopy with magnification is useful for detecting metachronous superficial pharyngeal cancer in patients with esophageal squamous cell carcinoma. J. Gastroenterol. Hepatol..

[85] Nakanishi H (2014). Detection of pharyngeal cancer in the overall population undergoing upper GI endoscopy by using narrow-band imaging: a single-center experience, 2009-2012. Gastrointest. Endosc..

[86] Li ZH (2013). The clinical utility of narrow band imaging in the surveillance of mucosa and sub-mucosa lesions in head and neck regions. Head Neck Oncol..

[87] Zabrodsky M, Lukes P, Lukesova E, Boucek J, Plzak J (2014). The role of narrow band imaging in the detection of recurrent laryngeal and hypopharyngeal cancer after curative radiotherapy. Biomed. Res. Int..

[88] Nguyen P (2013). High specificity of combined narrow band imaging and autofluorescence mucosal assessment of patients with head and neck cancer. Head Neck.

[89] Yang SW, Lee YS, Chang LC, Hwang CC, Chen TA (2014). Use of endoscopy with narrow-band imaging system in detecting squamous cell carcinoma in oral chronic non-healing ulcers. Clin. Oral Investig..

[90] Garofolo S (2015). Intraoperative narrow band imaging better delineates superficial resection margins during transoral laser microsurgery for early glottic cancer. Ann. Otol. Rhinol. Laryngol..

[91] Vicini C, Montevecchi F, D’Agostino G, DEV A, Meccariello G (2015). A novel approach emphasising intra-operative superficial margin enhancement of head-neck tumours with narrow-band imaging in transoral robotic surgery. Acta Otorhinolaryngol. Ital..

[92] Tirelli G, Piovesana M, Gatto A, Torelli L, Boscolo Nata F (2016). Is NBI-guided resection a breakthrough for achieving adequate resection margins in oral and oropharyngeal squamous cell carcinoma?. Ann. Otol. Rhinol. Laryngol..

[93] Louie JS, Shukla R, Richards-Kortum R, Anandasabapathy S (2015). High-resolution microendoscopy in differentiating neoplastic from non-neoplastic colorectal polyps. Best. Pract. Res. Clin. Gastroenterol..

[94] Protano MA (2015). Low-cost high-resolution microendoscopy for the detection of esophageal squamous cell neoplasia: an international trial. Gastroenterology.

[95] Pierce M, Yu D, Richards-Kortum R (2011). High-resolution fiber-optic microendoscopy for in situ cellular imaging. J. Vis. Exp.

[96] Zhong W (2009). In vivo high-resolution fluorescence microendoscopy for ovarian cancer detection and treatment monitoring. Br. J. Cancer.

[97] Dromard T, Ravaine V, Ravaine S, Leveque JL, Sojic N (2007). Remote in vivo imaging of human skin corneocytes by means of an optical fiber bundle. Rev. Sci. Instrum..

[98] Polglase AL (2005). A fluorescence confocal endomicroscope for in vivo microscopy of the upper- and the lower-GI tract. Gastrointest. Endosc..

[99] Bedard N, Quang T, Schmeler K, Richards-Kortum R, Tkaczyk TS (2012). Real-time video mosaicing with a high-resolution microendoscope. Biomed. Opt. Express.

[100] Levy LL (2016). OP078: High resolution microendoscopy of intraoperative resection margins in sqaumous cell carcinoma of the oral cavity. Oral Oncol..

[101] Miles BA (2015). Operative margin control with high-resolution optical microendoscopy for head and neck squamous cell carcinoma. Laryngoscope.

[102] Pierce MC (2012). Accuracy of in vivo multimodal optical imaging for detection of oral neoplasia. Cancer Prev. Res..

[103] Muldoon TJ (2012). Noninvasive imaging of oral neoplasia with a high-resolution fiber-optic microendoscope. Head Neck.

[104] Ishijima A (2015). Automated frame selection process for high-resolution microendoscopy. J. Biomed. Opt..

[105] Lloyd GR (2013). Discrimination between benign, primary and secondary malignancies in lymph nodes from the head and neck utilising Raman spectroscopy and multivariate analysis. Analyst.

[106] Li Y (2010). Research on the Raman spectral character and diagnostic value of squamous cell carcinoma of oral mucosa. J. Raman Spectrosc..

[107] Teh SK (2010). Near-infrared Raman spectroscopy for early diagnosis and typing of adenocarcinoma in the stomach. Br. J. Surg..

[108] Darlenski R, Fluhr JW (2016). In vivo Raman confocal spectroscopy in the investigation of the skin barrier. Curr. Probl. Dermatol..

[109] Zhao J, Lui H, Kalia S, Zeng H (2015). Real-time Raman spectroscopy for automatic in vivo skin cancer detection: an independent validation. Anal. Bioanal. Chem..

[110] Ashok PC (2013). Multi-modal approach using Raman spectroscopy and optical coherence tomography for the discrimination of colonic adenocarcinoma from normal colon. Biomed. Opt. Express.

[111] Chowdary MV (2007). Discrimination of normal and malignant mucosal tissues of the colon by Raman spectroscopy. Photomed. Laser Surg..

[112] Shi H, Chen SY, Lin K (2016). Raman spectroscopy for early real-time endoscopic optical diagnosis based on biochemical changes during the carcinogenesis of Barrett’s esophagus. World J. Gastrointest. Endosc..

[113] Bergholt MS (2014). Fiberoptic confocal raman spectroscopy for real-time in vivo diagnosis of dysplasia in Barrett’s esophagus. Gastroenterology.

[114] Li S (2015). Characterization and noninvasive diagnosis of bladder cancer with serum surface enhanced Raman spectroscopy and genetic algorithms. Sci. Rep..

[115] Canetta E (2014). Discrimination of bladder cancer cells from normal urothelial cells with high specificity and sensitivity: combined application of atomic force microscopy and modulated Raman spectroscopy. Acta Biomater..

[116] Shao X (2016). Evaluation of expressed prostatic secretion and serum using surface-enhanced Raman spectroscopy for the noninvasive detection of prostate cancer, a preliminary study. Nanomed.: Nanotechnol. Biol. Med..

[117] Del Mistro G (2015). Surface-enhanced Raman spectroscopy of urine for prostate cancer detection: a preliminary study. Anal. Bioanal. Chem..

[118] Santos IP (2017). Raman spectroscopy for cancer detection and cancer surgery guidance: translation to the clinics. Analyst.

[119] Stone N, Stavroulaki P, Kendall C, Birchall M, Barr H (2000). Raman spectroscopy for early detection of laryngeal malignancy: preliminary results. Laryngoscope.

[120] Lau DP (2005). Raman spectroscopy for optical diagnosis in the larynx: preliminary findings. Lasers Surg. Med..

[121] Guze K (2015). Pilot study: Raman spectroscopy in differentiating premalignant and malignant oral lesions from normal mucosa and benign lesions in humans. Head Neck.

[122] Singh SP, Deshmukh A, Chaturvedi P, Murali Krishna C (2012). In vivo Raman spectroscopic identification of premalignant lesions in oral buccal mucosa. J. Biomed. Opt..

[123] Oliveira AP, Bitar RA, Silveira L, Zangaro RA, Martin AA (2006). Near-infrared Raman spectroscopy for oral carcinoma diagnosis. Photomed. Laser Surg..

[124] Yan B (2011). Discrimination of parotid neoplasms from the normal parotid gland by use of Raman spectroscopy and support vector machine. Oral Oncol..

[125] Yan B (2015). Label-free blood serum detection by using surface-enhanced Raman spectroscopy and support vector machine for the preoperative diagnosis of parotid gland tumors. BMC Cancer.

[126] Cals FL (2015). Investigation of the potential of Raman spectroscopy for oral cancer detection in surgical margins. Lab. Invest..

[127] Cals FL (2016). Development and validation of Raman spectroscopic classification models to discriminate tongue squamous cell carcinoma from non-tumorous tissue. Oral Oncol..

[128] Barroso EM (2015). Discrimination between oral cancer and healthy tissue based on water content determined by Raman spectroscopy. Anal. Chem..

[129] Barroso EM (2016). Water concentration analysis by Raman spectroscopy to determine the location of the tumor border in oral cancer surgery. Cancer Res..

